# Mitochondrial and chloroplast genomes provide insights into the evolutionary origins of quinoa (*Chenopodium quinoa* Willd.)

**DOI:** 10.1038/s41598-018-36693-6

**Published:** 2019-01-17

**Authors:** Peter J. Maughan, Lindsay Chaney, Damien J. Lightfoot, Brian J. Cox, Mark Tester, Eric N. Jellen, David E. Jarvis

**Affiliations:** 1 Brigham Young University, Department of Plant and Wildlife Sciences, College of Life Sciences, Provo, Utah 84602 USA; 2 Snow College, Department of Biological Sciences, Division of Natural Science and Mathematics, Ephraim, Utah 84627 USA; 3 King Abdullah University of Science and Technology (KAUST), Biological and Environmental Sciences & Engineering Division (BESE), Thuwal, 23955-6900 Saudi Arabia

## Abstract

Quinoa has recently gained international attention because of its nutritious seeds, prompting the expansion of its cultivation into new areas in which it was not originally selected as a crop. Improving quinoa production in these areas will benefit from the introduction of advantageous traits from free-living relatives that are native to these, or similar, environments. As part of an ongoing effort to characterize the primary and secondary germplasm pools for quinoa, we report the complete mitochondrial and chloroplast genome sequences of quinoa accession PI 614886 and the identification of sequence variants in additional accessions from quinoa and related species. This is the first reported mitochondrial genome assembly in the genus *Chenopodium*. Inference of phylogenetic relationships among *Chenopodium* species based on mitochondrial and chloroplast variants supports the hypotheses that 1) the A-genome ancestor was the cytoplasmic donor in the original tetraploidization event, and 2) highland and coastal quinoas were independently domesticated.

## Introduction

Quinoa (*Chenopodium quinoa* Willd.) is an emerging pseudocereal crop whose recent increase in popularity is primarily due to its highly nutritious seeds, which contain higher protein levels and a more balanced amino acid profile than most of the grass-cereals^1^. Quinoa is native to the Andean and Mediterranean-coastal climate zones of South America and consequently possesses many desirable agronomic traits, such as tolerance to harsh abiotic conditions, including frost and high soil salinity^2^; however, many traits are not suitable for large-scale agricultural production, especially as global quinoa cultivation expands into non-native areas to which quinoa is not adapted. For example, many quinoa accessions are prone to lodging, susceptible to fungal pathogens, sensitive to heat, or excessively branched and therefore suboptimal for mechanized, irrigated cropping systems. Although it was domesticated thousands of years ago^3^, efforts to develop the genomic tools needed to improve quinoa only began within the last 20 years. High-quality genome assemblies are now available for accessions representing the two major ecotypes: PI 614886^4^ (coastal ecotype) and ‘Real’^5^ (highland or Salares ecotype). In addition, chloroplast genome assemblies are available for three quinoa accessions: ‘Real’^5^, PI 510550^6^, and PI 433232^7^. These reference genome sequences can now be used to identify variants in related accessions and species that might be useful in quinoa breeding, as well as to help elucidate the evolutionary history of quinoa.

Quinoa is an allotetraploid (2*n* = 4*x* = 36) which arose as the result of the hybridization between A- and B-sub-genome diploid species between 3.3 and 6.3 million years ago^4^, possibly in North America. The A and B sub-genomes are also shared by the related tetraploid species *C*. *berlandieri* Moq. and *C*. *hircinum* Schrad., which, together with quinoa, form an interfertile group known as the allotetraploid goosefoot complex (ATGC). Long-range dispersal of the ancestral free-living ATGC eventually resulted in the appearance and domestication of quinoa in the Lake Titicaca Basin over 7,000 years ago^8^. In addition to quinoa, the ATGC was the source of at least three other cultigens: Mesoamerican vegetable *huauzontle* and pseudocereal *chia roja* (*C*. *berlandieri* ssp. *nuttaliae*)^9^; and the extinct staple pseudocereal of the North American Hopewell Culture, *C*. *berlandieri* ssp. *jonesianum*^10^. Free-living *C*. *berlandieri* and *C*. *hircinum* occupy a much larger geographic distribution than quinoa^4^ and have therefore naturally adapted to many of the environments into which quinoa production is expanding; thus, these species, as well as sympatric free-living *C*. *quinoa* ssp. *melanospermum* (known by indigenous peoples as Ajara), represent potential sources of advantageous traits that can be bred into cultivated quinoa varieties.

As an important step in characterizing the primary and secondary germplasm pools for quinoa, we report the *de novo* assembly of the complete mitochondrial and chloroplast genomes of the coastal accession PI 614886 and the identification of sequence variants in 13 additional accessions of quinoa, five accessions of *C*. *berlandieri*, two accessions of *C*. *hircinum*, and the diploid species *C*. *pallidicaule* Aellen. and *C*. *suecicum* Murr. (representing the A and B sub-genomes, respectively). This is the first report of an assembled mitochondrial genome for the genus. We show evidence that the A-sub-genome ancestor of quinoa was likely the maternal parent in the tetraploidization event, supporting the hypothesis that the initial polyploidization event likely occurred in the New World.

## Results and Discussion

### Mitochondrial genome assembly, annotation, and comparison

Unlike chloroplast genomes, plant mitochondrial genomes are highly variable among different species. As such, it is not possible to assemble complete mitochondrial genomes using purely read-mapping approaches. Here we assembled previously reported^4^ Illumina sequencing reads with the Assembly by Reduced Complexity (ARC)^11^ assembly pipeline seeded with a reference mitochondrial target from *Beta vulgaris* L.^12^ (sugar beet) and gap-filled with long sequencing reads from Pacific Biosciences (PacBio) to produce the first reported mitochondrial genome assembly in quinoa. The assembly is 315,003 bp in length, which is shorter than the reported mitochondrial sequences of *B*. *vulgaris* (368,801 bp)^12^, *B*. *vulgaris* ssp. *maritima* (sea beet; 364,950 bp), and *Spinacia oleracea* L. (spinach; 329,613 bp)^13^ – the only other species in the Amaranthaceae with reported mitochondrial sequences.

**Figure 1:**
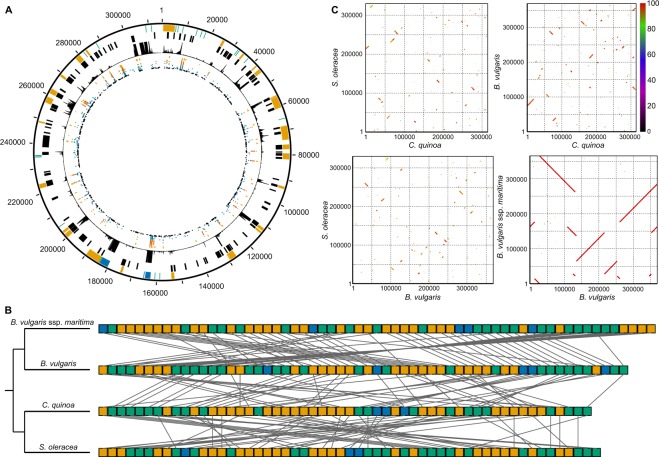
Structure of the mitochondrial genome in quinoa and related species. (**A**) Features of the mitochondrial genome assemblies in quinoa. Starting from the outside, tracks represent position (bp), annotated genes (orange, CDS; blue, tRNA; green, rRNA), predicted ORFs, RNA-seq read depth, and SNPs (black, *C*. *pallidicaule* and *C*. *suecicum*; blue, *C*. *berlandieri*; green, *C*. *hircinum*; brown, highland quinoa; orange, coastal quinoa). (**B**) Linear order of genes (orange, CDS; blue, tRNA; green, rRNA) in the mitochondrial genomes of quinoa and related species. Species are arranged according to the accepted organismal phylogeny, with lines connecting genes with the same annotation. Boxes are not proportional to actual gene length. (**C**) Dotplot visualization of nucleotide-by-nucleotide comparisons of the mitochondrial sequence from Amaranthaceae species. Axes are reported in bp. Scale bar reports percent nucleotide identity.

The assembled mitochondrial sequence was annotated with 30 protein-coding genes, 21 tRNA genes, and 3 rRNA genes (Fig. 1a; Table 1), which is similar to the 29 and 29 protein-coding genes, 25 and 24 tRNA genes, and 5 and 3 rRNA genes reported in the mitochondrial genomes of *B*. *vulgaris*^12^ and *S*. *oleracea*^13^, respectively. Most annotated genes are supported by strong evidence of expression (Fig. 1a). Twenty-eight (93.3%) of the protein-coding genes overlap with predicted open reading frames (ORFs) (Fig. 1a). A majority of the predicted ORFs (73 out of 112; 65.2%) do not overlap with any annotated genes; however, most of these ORFs show no evidence of expression, suggesting that they are false predictions (Fig. 1a). The quinoa mitochondrial sequence does not lack any genes common to the other sequenced Amaranthaceae mitochondrial genomes (Table 1), suggesting that the sequence is likely complete.

**Table 1:** Protein-coding, tRNA, and rRNA genes annotated in the quinoa mitochondrial genome, in comparison to related species.

Gene	*B*. *vulgaris* ssp. *maritima*	*B*. *vulgaris*	*C*. *quinoa*	*S*. *oleracea*
atp1	+	+	+	+
atp4	−	−	+	+
atp6	+	+	+	+
atp8	+	+	+	+
atp9	+	+	+	+
ccmB/ccb206	+	+	+	+
ccmC	−	−	+	+
ccmFC/ccb438	+	+	+	+
ccmFN/ccb577	+	+	+	+
cob	+	+	+	+
cox1	+	+	+	+
cox2	+	+	+	+
cox3	+	+	+	+
mat-r	+	+	+	+
nad1	++	+	+	−
nad2	+	+	+	+
nad3	+	+	+	+
nad4	+	+	+	+
nad4L	+	+	+	+
nad5	+	+	+	+
nad6	+	+	+	+
nad7	+	+	+	+
nad9	+	+	+	+
petG	+	−	−	−
rpl5	+	+	+	+
rps12	+	+	+	+
rps13	+	+	+	+
rps3	+	+	+	+
rps4	+	+	+	+
rps7	++	+	+	+
sdh4	+	−	−	−
tatC	+	−	+	+
rrn5	+	+	+	+
rrn18	+	+	+	+
rrn26	+++	+++	+	+
tRNA-Asn	+	+	+	+
tRNA-Asp	+	+	+	+
tRNA-Cys	+++	+++	++	++
tRNA-Gln	+	+	+	+
tRNA-Glu	+	+	+	+
tRNA-Gly	+	+	+	+
tRNA-His	+	+	+	++
tRNA-Ile	+	++	−	+
tRNA-Leu	−	−	−	+
tRNA-Lys	+	+	+	+
tRNA-Met	+++++	+++++	+++++	+++
tRNA-Phe	+	+	+	+++
tRNA-Pro	+	++	+	+
tRNA-Ser	+++	+++	+++	++
tRNA-Trp	+	+	+	+
tRNA-Tyr	+	+	+	+
tRNA-Val	+	+	−	−

^+^The number of copies of a gene present in each mitochondrial genome.

^−^The absence of a gene.

The quinoa mitochondrial genome was annotated with one copy each of the 5S, 18S, and 26S rRNA genes (Fig. 1a, Table 1). Additionally, a region of the mitochondrial sequence from approximately 305–308 kb was annotated as containing a fragment of the 26S rRNA gene, and BLAST analysis of this region showed high sequence similarity to the 26S rRNA gene from several other species, including *B*. *vulgaris*, which contains three annotated 26S rRNA genes. This region shows extremely high levels of expression that is representative of the expression seen from the other annotated rRNA genes (Fig. 1a). Thus, this sequence likely represents a second, possibly fragmented copy of the 26S rRNA gene which is still expressed in quinoa.

The quinoa mitochondrial genome also contains two tRNA^Cys^ genes (*trnC*-GCA): one copy, located near position 8.4 kb, is homologous to the native *trnC1*-GCA gene from *B*. *vulgaris*; the other copy, located near position 311.1 kb, is homologous to the two novel copies of *trnC2*-GCA from *B*. *vulgaris*. The *trnC2*-GCA gene from *B*. *vulgaris* had not previously been identified in other higher plants^12^; its presence in the quinoa mitochondrial genome suggests that this gene is shared among Amaranthaceae species.

Although there are few differences in gene content among the sequenced Amaranthaceae mitochondrial genomes (Table 1), there are substantial differences in gene order (Fig. 1b). Not surprisingly, there is greater conservation of gene order within species (for example, between *B*. *vulgaris* and *B*. *vulgaris* ssp. *maritima*) than between species (for example, between quinoa and *B*. *vulgaris* or *S*. *oleracea*). Similar patterns were also observed at the overall nucleotide level, in which a relatively small number of rearrangements have shuffled large sequences of DNA within *B*. *vulgaris*, but a much higher number of rearrangements have resulted in low levels of sequence collinearity between quinoa and the other Amaranthaceae species (Fig. 1c). Such extensive rearrangement has been observed in the mitochondrial genomes of other plant families^14^ but is in contrast to the high degree of conservation observed in mitochondrial genomes of animals^15,16^.

To assess variation within *Chenopodium*, re-sequencing data from 13 additional quinoa accessions; five and two accessions of the related tetraploid species *C*. *berlandieri* and *C*. *hircinum*, respectively; and one accession each of the A-genome diploid *C*. *pallidicaule* and the B-genome diploid *C*. *suecicum* were mapped onto the reference PI 614886 mitochondria assembly. Single nucleotide polymorphisms (SNPs) and insertion/deletion variants (InDels) were identified in each re-sequencing accession relative to the PI 614886 reference assembly (Table 2). As expected, more variants in the mitochondria were identified in the diploid species *C*. *pallidicaule* and *C*. *suecicum* (491 and 626 SNPs, and 188 and 203 InDels, respectively) than in the tetraploids (with an average of 90 SNPs and 83 InDels). We acknowledge that sequence rearrangements such as those described above can affect the efficiency of read mapping and variant calling; however, these rearrangements, which we anticipate to be few in number within *Chenopodium*, are difficult to identify using re-sequencing data.

**Table 2:** Variants in the mitochondrial and chloroplast genomes of *Chenopodium* species.

Species	Accession	Mitochondria		Chloroplast	
		SNP	INDEL	SNP	INDEL
*C*. *quinoa*	0654	109	91	69	28
*C*. *quinoa*	Cherry Vanilla	72	87	33	23
*C*. *quinoa*	Chucapaca	46	72	16	37
*C*. *quinoa*	CICA-17	60	100	36	23
*C*. *quinoa*	G-205-95DK	84	66	0	0
*C*. *quinoa*	Ku-2	90	72	0	0
*C*. *quinoa*	Kurmi	83	91	36	24
*C*. *quinoa*	Ollague	73	83	35	23
*C*. *quinoa*	Pasankalla	70	81	25	13
*C*. *quinoa*	PI 634921	56	65	17	36
*C*. *quinoa*	Real	78	90	37	24
*C*. *quinoa*	Regalona	85	68	3	0
*C*. *quinoa*	Salcedo INIA	74	87	33	23
*C*. *berlandieri* subsp. *nuttalliae*	PI 568156	198	97	63	30
*C*. *berlandieri* var. *boscianum*	BYU 937	151	95	52	29
*C*. *berlandieri* var. *macrocalycium*	PI 666279	85	70	60	28
*C*. *berlandieri* var. *sinuatum*	Ames 33013	146	89	50	30
*C*. *berlandieri* var. *zschackei*	BYU 1314	86	81	73	33
*C*. *hircinum*	BYU 1101	74	80	54	21
*C*. *hircinum*	BYU 566	72	86	25	14
*C*. *pallidicaule*	Ames 13221	491	188	709	145
*C*. *suecicum*	328/6	626	203	1181	221

Consensus sequences were generated from the mapped reads for each re-sequenced accession, and simple sequence repeats (SSRs) were identified in the consensus sequences as well as in the PI 614886 reference sequence. Similar numbers of SSRs were identified in all accessions, with 50 SSR motifs identified in PI 614886 and an average of 48 SSR motifs identified in each accession (Supplementary Table S2). Approximately half of all repeats in each accession are tetranucleotide repeats; no hexanucleotide repeats were identified in any of the accessions (Supplementary Table S2).

### Chloroplast genome assembly, annotation, and comparison

**Figure 2:**
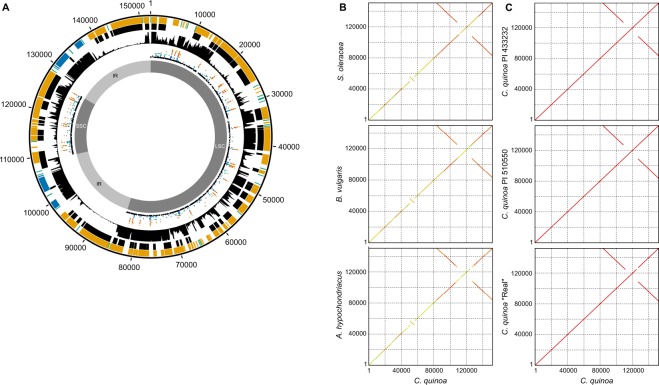
Structure of the chloroplast genome in quinoa and related species. (**A**) Features of the chloroplast genome assemblies in quinoa. Starting from the outside, tracks represent position (bp), annotated genes (orange, CDS; blue, tRNA; green, rRNA), predicted ORFs, RNA-seq read depth, SNPs (black, *C*. *pallidicaule* and *C*. *suecicum*; blue, *C*. *berlandieri*; green, *C*. *hircinum*; brown, highland quinoa; orange, coastal quinoa); and the positions of the LSC, SSC, and IR regions. (**B**,**C**) Dotplot visualization of nucleotide-by-nucleotide comparisons of the chloroplast sequence from Amaranthaceae species (**B**) and quinoa accessions (**C**). Axes are reported in bp.

We also used previously reported^4^ Illumina and PacBio reads to assemble the chloroplast genome of quinoa accession PI 614886 into a single circular contig 152,079 bp in length, which is almost identical to the lengths of chloroplast assemblies reported in other quinoa accessions (152,075 in PI 510550^6^; 152,099 in PI 433232^7^; and 152,282 in ‘Real’^5^), and slightly longer than those reported in other closely related Amaranthaceae species (149,635 in *B*. *vulgaris*^17^; 150,518 in *A*. *hypochondriacus*^18^; and 150,725 in *S*. *oleracea*^19^). The assembly contains the large (LSC) and small (SSC) single-copy regions separated by two inverted repeats (IR) that typically characterize chloroplast sequences (Fig. 2a).

The sequence was annotated with 86 protein-coding, 31 tRNA, and 8 rRNA genes (Fig. 2a, Table 3). Most annotated genes are supported by strong evidence of expression (Fig. 2a); however, only 40 (46.5%) of the protein-coding genes overlap perfectly with predicted ORFs, whereas 30 (34.9%) genes do not overlap with any predicted ORFs (Fig. 2a). Most (27) of the genes in the latter category, however, are shorter than the minimum length used for ORF prediction (300 bp). One of the three genes longer than 300 bp that does not overlap with any predicted ORFs, *atpF*, contains an intron, which may explain the absence of a predicted ORF. The other two genes are two copies of *rps12* located in the inverted repeats near positions 96.0 kb and 138.8 kb. These genes appear to be transpliced with a separate mRNA transcript produced from a fragment of *rps12* located at approximately 68.9 kb. Nine genes (10.5%) overlap only partially with predicted ORFs. In some cases, such as for *ndhA* and *rpl16*, this is likely due to the presence of introns disrupting the ORF. In other cases, such as for *ndhD* and *psbC*, it is due to the use of alternative translational start codons. At least one gene, *rpl23*, appears to be a pseudogene. Two sequences homologous to *rpl23* are present in the inverted repeats of the quinoa chloroplast genome at positions 84.5 kb and 150.8 kb. However, both sequences contain internal stop codons; hence, no open reading frames longer than 300 bp were predicted in these regions (Fig. 2a). Pseudogenization of *rpl23* has been reported in the chloroplast genomes of quinoa and other Amaranthaceae species^6,20^.

**Table 3:** Protein-coding, tRNA, and rRNA genes annotated in the quinoa chloroplast genome, in comparison to related species.

Gene	*A*. *hypochondriacus*	*B*. *vulgaris*	*C*. *quinoa*	*S*. *oleracea*
accD	+	+	+	+
atpA	+	+	+	+
atpB	+	−	+	+
atpE	+	+	+	+
atpF	+	+	+	+
atpH	+	+	+	+
atpI	+	+	+	+
ccsA	+	+	+	−
cemA	+	+	+	+
clpP	+	+	+	+
infA	+	+	+	+
matK	+	+	+	−
nad2	−	+	−	−
ndhA	+	+	+	+
ndhB	+	++	++	++
ndhC	+	+	+	+
ndhD	+	+	+	+
ndhE	+	+	+	+
ndhF	+	+	+	+
ndhG	+	+	+	+
ndhH	+	+	+	+
ndhI	+	+	+	+
ndhJ	+	+	+	+
ndhK	+	+	+	+
petA	+	+	+	+
petB	−	+	+	+
petD	+	+	+	+
petG	+	+	+	+
petL	+	+	+	+
petN	+	+	+	−
psaA	+	+	+	+
psaB	+	−	+	+
psaC	+	+	+	+
psaI	+	+	+	+
psaJ	+	+	+	+
psbA	+	+	+	+
psbB	+	−	+	+
psbC	+	+	+	+
psbD	+	+	+	+
psbE	+	+	+	+
psbF	+	+	+	+
psbH	+	+	+	+
psbI	+	+	+	+
psbJ	+	+	+	+
psbK	+	+	+	+
psbL	−	+	+	+
psbM	+	+	+	+
psbN/pbf1	+	+	+	+
psbT	+	+	+	+
psbZ	+	+	+	−
psi	−	+	−	−
rbcL	+	+	+	+
rpl14	+	+	+	+
rpl16	++	+	+	+
rpl2	+	++	++	+
rpl20	+	+	+	+
rpl22	+	+	+	+
rpl23	−	++	++	−
rpl32	+	+	+	+
rpl33	++	+	+	+
rpl36	−	−	+	+
rpoA	+	+	+	+
rpoB	+	+	+	+
rpoC1	+	+	+	+
rpoC2	+	+	+	+
rps2	+	+	+	+
rps12	++	−	++	++
rps14	−	+	+	+
rps15	+	+	+	+
rps16	+	+	+	+
rps18	+	+	+	+
rps19	−	+	+	+
rps2	+	+	+	+
rps3	+	+	+	+
rps4	+	+	+	+
rps7	+	++	++	++
rps8	+	+	+	+
ycf1	−	++	+	+
ycf2	−	++	++	+
ycf3	−	+	+	++
ycf4	+	+	+	+
ycf5	−	−	−	+
ycf6	−	−	−	+
ycf9	−	−	−	+
tRNA-Ala	++	++	++	++
tRNA-Arg	+++	+++	+++	+++
tRNA-Asn	++	++	++	++
tRNA-Asp	+	+	+	+
tRNA-Cys	+	+	+	+
tRNA-Gln	+	+	+	+
tRNA-Glu	+	+	+	+
tRNA-Gly	+	++	+	++
tRNA-His	+	+	+	+
tRNA-Ile	−	++++	−	++++
tRNA-Leu	+++	++++	+++	++++
tRNA-Lys	−	+	−	+
tRNA-Met	++++	++	++++	++
tRNA-Phe	+	+	+	+
tRNA-Pro	+	+	+	+
tRNA-Ser	+++	+++	+++	+++
tRNA-Thr	++	++	++	++
tRNA-Trp	+	+	+	+
tRNA-Tyr	+	+	+	+
tRNA-Val	++	+++	++	+++
rrn4.5	++	++	++	++
rrn16	++	++	++	++
rrn23	++	++	++	++
rrn5	++	++	++	++

^+^The number of copies of a gene present in each chloroplast genome.

^−^The absence of a gene.

The quinoa chloroplast sequence does not lack any genes common to the *A*. *hypochondriacus*, *B*. *vulgaris*, and *S*. *oleracea* chloroplast genomes, nor does it have any unique genes not found in the chloroplast sequences of these species. Indeed, there is a high degree of sequence conservation among chloroplast genomes of the Amaranthaceae species, with the main structural difference between quinoa and the other species being a small inversion (Fig. 2b) that has been previously reported in quinoa^6^. No major structural variations were observed among all the quinoa chloroplast genome assemblies (Fig. 2c), suggesting that the observed inversion relative to other Amaranthaceae species is not due to an assembly error. Despite the absence of major structural variation among the quinoa accessions, dotplot visualization of nucleotide-level comparisons among the quinoa chloroplast assemblies revealed a region near position 125 kb in ‘Real’ that did not match the corresponding region in PI 614886 (Fig. 2c, bottom panel). A BLAST analysis of each previously reported quinoa assembly against the PI 614886 assembly confirmed that the non-homologous sequence in this region was found only in the ‘Real’ assembly (Supplementary Fig. S1a). This region is within the predicted *ycf1* gene. A BLAST search of the *ycf1* sequence from *Arabidopsis thaliana* indicated that the *ycf1* gene is complete in the PI 614886 assembly (Supplementary Fig. S1b, top track) but is missing approximately 2 kb of sequence in the middle of the gene in the ‘Real’ assembly (Supplementary Fig. S1b, middle track). Additional BLAST searches indicated that the ‘Real’ assembly contains the *ndhF* gene nested within *ycf1*. This *ndhF* gene was not identified in the consensus sequence (Supplementary Fig. S1b, bottom track) or the mapped raw reads (Supplementary Fig. S1a, inner track) of our re-sequenced ‘Real’ accession. The ‘Real’ accession is more appropriately considered an ecotype, as it represents a collection of landraces from the Salares region of the Bolivian Altiplano. Thus, although the discrepancy between the published ‘Real’ chloroplast assembly and our re-sequencing data may be due to assembly errors, it is also possible that it represents sequence variation among ‘Real’ genotypes.

Data from the re-sequenced accessions was mapped onto the quinoa reference (PI 614886) chloroplast assembly, as was done with the mitochondrial sequence, and SNPs and InDels were identified in each re-sequencing accession relative to the PI 614886 reference assembly (Table 2). As was observed with the mitochondria, more variants were identified in the diploid species *C*. *pallidicaule* and *C*. *suecicum* (709 and 1181 SNPs, and 145 and 221 InDels, respectively) than in the tetraploids (with an average of 36 SNPs and 22 InDels). Collectively, 301 and 1,650 unique SNP positions were identified in the tetraploids and diploids, respectively; 56 (19%) of the tetraploid SNP positions also contained SNPs in at least one of the diploids, whereas 42 (3%) of the diploid SNP positions also contained SNPs in at least one of the tetraploids (Fig. 2a; to avoid confounding results, SNPs identified in the highly similar IR regions were excluded). No SNPs and/or very few InDels were identified in the chloroplast sequences of three quinoa accessions: ‘G-205-95DK’, ‘Ku-2’, and ‘Regalona’. All three accessions and the reference accession PI 614886 are of the coastal type (Supplementary Table S1), which may explain the low level of chloroplast sequence variation in these accessions.

As was done for the mitochondria, consensus chloroplast sequences were generated from the mapped reads for each re-sequenced accession, and simple sequence repeats (SSRs) were identified in the consensus PI 614886 reference chloroplast sequence. Similar numbers of SSRs were identified in all accessions, with 14 SSR motifs identified in PI 614886 and an average of 15 SSR motifs in each accession (Supplementary Table S3). As with the mitochondria, tetranucleotide repeats were the most common type identified in the chloroplast, making up approximately 40% of all repeats. Two accessions of *C*. *berlandieri* contained hexanucleotide repeats.

### Phylogenetic relationships

**Figure 3:**
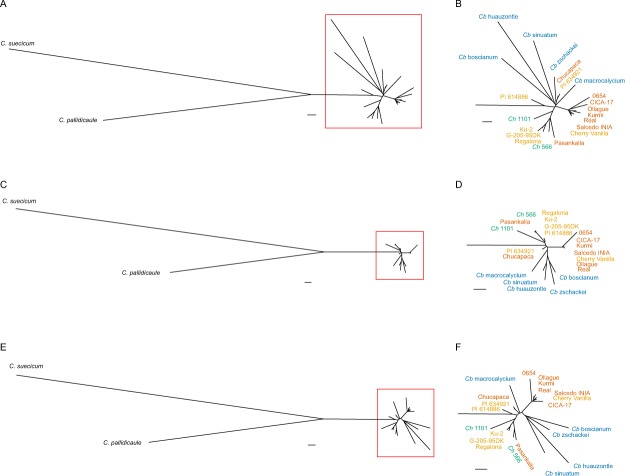
Phylogenetic relationships among *Chenopodium* species based on variants in the mitochondria (**A**,**B**), chloroplast (**C**,**D**), and combined mitochondria/chloroplast (**E**,**F**). Networks displayed in (**B**), (**D**), and (**F**) are enlarged from the red-boxed regions shown in (**A**), (**C**), and (**E**), respectively. Black text, *C*. *pallidicaule* and *C*. *suecicum*; blue text, *C*. *berlandieri* (*Cb*); green text, *C*. *hircinum* (*Ch*); brown, highland quinoa; orange, coastal quinoa. Scale bar, 0.01 substitutions per site.

Phylogenetic relationships among *Chenopodium* species based on variants in the mitochondria (Fig. 3a,b), chloroplast (Fig. 3c,d), and combined mitochondria/chloroplast (Fig. 3e,f) illustrate a number of relationships that are concordant with previous analyses of nuclear genome sequence data^4^. The two diploid species, *C*. *pallidicaule* (A-genome group) and *C*. *suecicum* (B-genome group) form the ancestral root of the tree from which the AABB ATGC tetraploids are derived. The A-genome ancestor is closer to the tetraploids, suggesting it was the cytoplasmic donor – and hence the likely maternal parent – in the original polyploidization event. Various accessions of *C*. *berlandieri* (blue text), the North American free-living species, are positioned more basally relative to the cultivated South American accessions of quinoa and the two accessions of the free-living South American taxon *C*. *hircinum* (green text). In general, the quinoas fall into two main groups: highland Andean (brown text) and a more distant branch consisting mostly of coastal ecotypes (orange text) as well as the Altiplano variety ‘Pasankalla’ and the two *C*. *hircinum* accessions. The distal placement of *C*. *hircinum* accessions on the coastal branch in all three trees suggests these are not ancestral to the Andean quinoas and may be representative of either ancestral or introgressive relationships with the coastal quinoa germplasm, as was also observed in the nuclear genome-based analysis^4^. Consequently, cytoplasmic DNA sequencing provides additional support for the hypothesis of separate Andean and Pacific coastal domestication events of South American goosefoot/quinoa.

Interestingly, the Andean variety Chucapaca and the coastal line PI 634921 (origin, Valdivia, Los Rios Region, Chile) were both positioned basally with accessions of *C*. *berlandieri* instead of with other accessions within their respective ecotype clades. It is tempting to speculate that introgression occurred with *C*. *berlandieri* at some point in their histories; however, nuclear genome analysis clearly groups these lines with other quinoas of their respective ecotypes^4^.

## Methods

### Mitochondrial genome assembly and annotation

We used the Assembly by Reduced Complexity (ARC)^11^ pipeline seeded with a reference mitochondrial target from *B*. *vulgaris* to assemble the mitochondrial genome of quinoa accession PI 614886. In brief, the ARC assembly pipeline used Bowtie2^21^ to align 12 million previously reported^4^ Illumina read pairs to a *B*. *vulgaris* mitochondrial target reference (NC_002511.2). Reads with high quality matches to the reference were extracted and assembled into quinoa-specific contigs using the SPAdes assembler^22^. The ARC pipeline was then used to extend the assembled quinoa mitochondrial contigs using four (numcycles = 4) successive rounds of mapping and re-assembly. Since mitochondria read depth should be significantly higher than nuclear genome read depth, only contigs with read depth >20 × (n = 18) were selected for gap-filling and circularization. PacBio long reads greater than 15 kb in length (n = 100,000) were used to fill gaps between contigs using the program PBJelly, a subprogram of PBSuite^23^. Visual inspection of the linear genome identified overlapping edges which were then stitched together to produce the final circular mitochondrial genome. Lastly, the mitrochondrial assembly was polished by re-mapping the same 12 million Illumina read pairs using the CLC Genomics Workbench, removing all unpaired reads (which likely occurred due to homology with nuclear genome sequences), and generating a final consensus sequence.

A consensus-corrected mitochondrial genome for each of the previously sequenced^4^ accessions of quinoa, *C*. *berlandieri*, *C*. *hircinum*, *C*. *pallidicaule*, and *C*. *suecicum* (Supplementary Table S1) was developed by mapping 12 million paired-end Illumina reads to the reference quinoa PI 614886 mitochondrial genome using the CLC Genomics Workbench and default parameters, with the exception that the length fraction and similar fractions were increased to 0.7 and 0.9, respectively. The mapped reads were then realigned to improve local alignment, followed by majority rule consensus sequence extraction for each accession.

The assembled quinoa mitochondrial genome was annotated using GeSeq^24^, selecting the options to perform tRNAscan-SE and BLAT using the NCBI RefSeq sequences for *B*. *vulgaris* and *S*. *oleracea*. ORFs with a minimum length of 300 bp were predicted using ORFfinder from NCBI^25^.

### Chloroplast genome assembly and annotation

We identified in the previously published genome assembly of the quinoa accession PI 614886^4^ a contig (unitig_5592) that consisted of a single contiguous sequence completely encompassing the IRa, SSC, and IRb regions of the chloroplast genome, as well as significant portions of the LSC region. The missing portion of the LSC was assembled using previously reported^4^ Illumina short reads and the ARC assembly pipeline seeded with the *S*. *oleracea* chloroplast genome sequence (NC_002202.1), as described above for the mitochondrial assembly. The ARC assembly pipeline produced a single contig which encompassed the missing LSC region (82,805 bp) that overlapped both ends of unitig_5592 which when spliced together produced a single circular molecule representing the complete quinoa chloroplast genome.

A consensus-corrected chloroplast genome for each of the related accessions (Supplementary Table S1) was developed by mapping 6 million paired-end Illumina reads to the reference quinoa chloroplast genome using the CLC Genomics Workbench, as described above for the mitochondrial genome.

The assembled quinoa chloroplast genome was annotated using GeSeq, selecting the options to perform HMMER profile search (chloroplast CDS and rRNA), tRNAscan-SE, and BLAST using the MPI-MP chloroplast references, while leaving all other default settings. ORFs with a minimum length of 300 bp were predicted using ORFfinder.

### Identification of SNPs, InDels, and SSRs

Microsatellites, or simple sequence repeats (SSRs), were identified using MISA^26^. We followed standard thresholds for identifying chloroplast and mitochondrial SSRs^27,28^, specifically a minimum stretch of 12 for mono-, 6 for di-, 4 for tri- and 3 for tetra-, penta- and hexa-nucleotide repeats and a minimum distance of 100 bp between compound SSRs.

SNPs and InDels were identified by aligning reads to the reference genome using BWA-MEM^29^ for each of the accessions. SNPs and InDels were identified using the Genome Analysis Tool Kit HaplotypeCaller (GATK-HC)^30^. A small number of SNPs were identified when mapping reads of PI 614886 to the reference PI 614886 assemblies, mainly because of the mapping of single, unpaired reads that likely had homology with nuclear sequences. Because these positions do not represent true variants, all positions for which a SNP was identified in PI 614886 were removed from variant calls in all other accessions.

### Genome visualization

Circular visualization of the chloroplast and mitochondrial genome assemblies of PI 614886 was performed using Circa. CDS, tRNA, and rRNA features were plotted from the GeSeq annotations, and ORF features were plotted from the ORFfinder predictions, as described above. Read depth was calculated from RNA-seq reads generated from several quinoa tissues, as previously described^4^. Briefly, reads were trimmed using Trimmomatic^31^ and mapped to the PI 614886 chloroplast and mitochondrial genome assemblies using TopHat^32^. Read depth at each nucleotide position was calculated using SAMtools mpileup. SNP positions in the re-sequenced accessions were plotted from the variant calling files, as described above.

### Comparison to previously sequenced genomes

Previously reported mitochondrial assemblies were downloaded for *B*. *vulgaris* subsp. *vulgari*^12^ (GenBank accession NC_002511.2), *B*. *vulgaris* subsp. *maritima* (Genbank accession NC_015099.1) and *S*. *olerace*^13^ (GenBank accession NC_035618.1). The genomes were compared using the default settings in nucmer from the mummer package^33^.

Previously published chloroplast assemblies were downloaded for *A*. *hypochondriacus*^18^ (GenBank accession NC_030770.1), *B*. *vulgaris*^17^ (GenBank accession KJ081864.1), and *S*. *oleracea*^19^ (GenBank accession AJ400848.1), and for quinoa accessions PI 433232^7^ (GenBank accession KY419706), PI 510550^6^ (GenBank accession KY635884), and ‘Real’^5^ (contig NSDK01003185.1 from GenBank accession NSDK00000000.1). Nucleotide-level comparisons of these genomes were performed using the default settings in nucmer. Further comparisons of the previously published quinoa chloroplast genomes to that of PI 614886 were performed using the CGView Comparison Tool^34^. In order to conduct an appropriate comparison of these genome sequences, each was annotated using GeSeq, as described above. The *A*. *thaliana ycf1* (GenBank accession NC_000932.1, nucleotides 129244-123884) and *ndhF* (Genbank accession NC_000932.1, nucleotides 112638-110398) genes were compared to the PI 614886 and ‘Real’ chloroplast assemblies, as well as to the ‘Real’ consensus sequence reported herein, using BLASTn. Results were visualized using Kablammo^35^, showing only hits with *e*-value < 1e-10.

### Phylogenetic network analysis

VCF files created by mapping re-sequencing reads to the chloroplast and mitochondrial genomes, as described above, were filtered with custom Perl scripts to identify SNPs with quality scores of at least 200. Phylogenetic networks for the chloroplast, mitochondrial, and combined chloroplast and mitochondrial SNPs were then computed with SplitsTree^36^ using an uncorrected P distance algorithm and the Neighbor-Net neighboring network.

## Electronic supplementary material

Supplementary Information

## Data Availability

All raw data were previously reported^4^. The quinoa chloroplast and mitochondrial genome assemblies and annotations have been uploaded to NCBI under GenBank accession numbers MK159176 and MK182703.
