# Supplementary material for: Mitochondrial and chloroplast genomes provide insights into the evolutionary origins of quinoa (Chenopodium quinoa Willd.)
Source: Sci Rep. 2019 Jan 17;9:185. doi: 10.1038/s41598-018-36693-6 (PMC6336861; doi:10.1038/s41598-018-36693-6)
Supplement: Supplementary file 1 — Supplementary Information [file 41598_2018_36693_MOESM1_ESM.pdf]

Mitochondrial and chloroplast genomes provide insights into the evolutionary origins of quinoa (*Chenopodium quinoa* Willd.)

Peter J. Maughan, Lindsay Chaney, Damien J. Lightfoot, Brian J. Cox, Mark Tester, Eric N. Jellen, David E. Jarvis

Supplementary Figure 1. Comparison of quinoa chloroplast assemblies. (A) Circular representation of BLAST sequence similarity of previously reported complete chloroplast assemblies relative to that of PI 614886. From the outside, tracks represent annotated genes in PI 614886 in the forward and reverse directions, similarity of PI 510550, similarity of PI 433232, similarity of ‘Real’, and sequencing read depth of the re-sequenced ‘Real’ accession. (B) BLAST sequence similarity of the chloroplasts of the PI 614886 assembly, the ‘Real’ assembly, and the ‘Real’ consensus sequence based on re-sequencing data, compared to *Arabidopsis ycf1* (blue) and *Arabidopsis ndhF* (red).

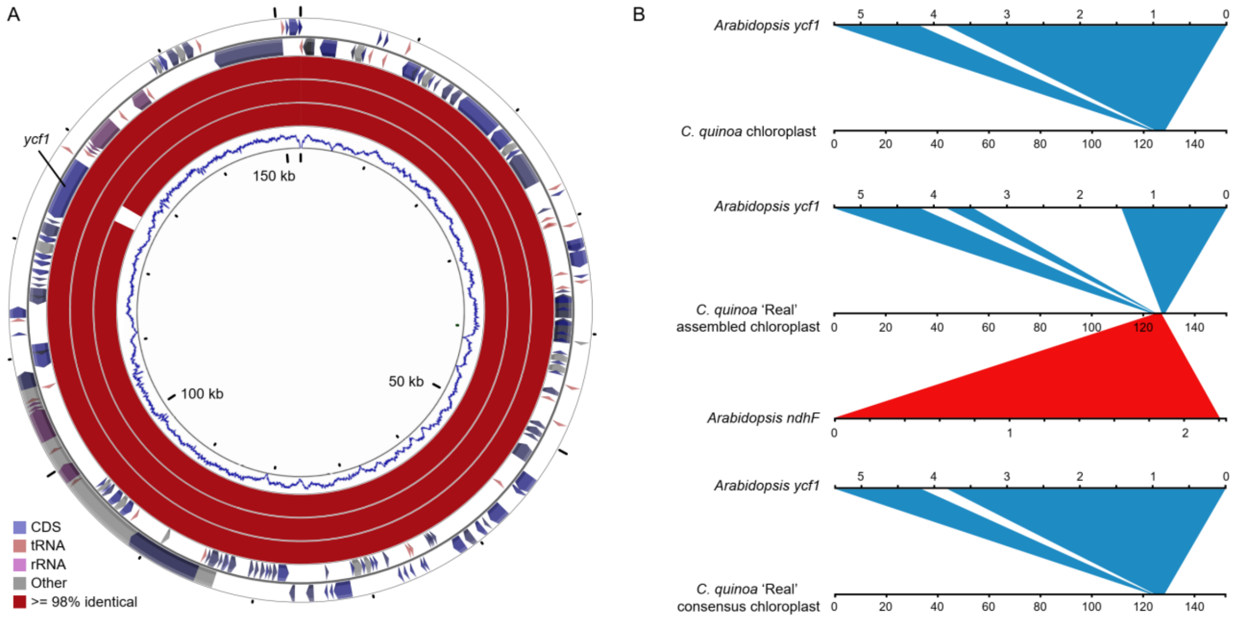

Supplementary Table S1. Species used in this study.

Species	Accession	Origin	Type	Ecotype	Ploidy	Source
C. quinoa	0654	Peru	Highland	Altiplano	$2n=4x=36$	CIP-FAO
C. quinoa	Cherry Vanilla	Oregon, US	Coastal	Coastal	$2n=4x=36$	Wild Garden Seed
C. quinoa	Chucapaca	Bolivia	Highland	Altiplano	$2n=4x=36$	CIP-FAO
C. quinoa	CICA-17	Peru	Highland	Andean Valley	$2n=4x=36$	CIP-FAO
C. quinoa	G-205-95DK	Denmark	Coastal	Coastal	$2n=4x=36$	CIP-FAO
C. quinoa	Ku-2	Chile	Coastal	Coastal	$2n=4x=36$	CIP-FAO
C. quinoa	Kurmi	Bolivia	Highland	Altiplano	$2n=4x=36$	PROINPA
C. quinoa	Ollague	Chile	Highland	Salares	$2n=4x=36$	CIP-FAO

C. quinoa	Pasankalla	Peru	Highland	Andean Valley	2*n*=4*x*=36	BYU Collection
C. quinoa	PI 614886	Chile	Coastal	Coastal	2*n*=4*x*=36	USDA-NPGS
C. quinoa	PI 634921	Chile	Coastal	Coastal	2*n*=4*x*=36	USDA-NPGS
C. quinoa	Real	Bolivia	Highland	Salares	2*n*=4*x*=36	CIP-FAO
C. quinoa	Regalona	Chile	Coastal	Coastal	2*n*=4*x*=36	Semillas Baer
C. quinoa	Salcedo INIA	Peru	Highland	Altiplano	2*n*=4*x*=36	CIP-FAO
C. berlandieri subsp. *nuttalliae*	PI 568156	Mexico		Cultivated (huauzontle)	2*n*=4*x*=36	USDA-NPGS
C. berlandieri var. *boscianum*	BYU 937	Texas, US		Wild/weedy	2*n*=4*x*=36	BYU Collection
C. berlandieri var. *macrocalycium*	PI 666279	Maine, US		Wild/weedy	2*n*=4*x*=36	USDA-NPGS
C. berlandieri var. *sinuatum*	Ames 33013	Arizona, US		Wild/weedy	2*n*=4*x*=36	USDA-NPGS
C. berlandieri var. *zschackei*	BYU 1314	Utah, US		Wild/weedy	2*n*=4*x*=36	BYU Collection
C. hircinum	BYU 1101	Argentina		Weedy (pampas)	2*n*=4*x*=36	Daniel Bertero
C. hircinum	BYU 566	Chile		Weedy (desert valley)	2*n*=4*x*=36	Francisco Fuentes
C. pallidicaule	Ames 13221	Bolivia		Cultivated (kaniwa)	2*n*=2*x*=18	USDA-NPGS
C. suecicum	328/6	Czech Republic		Weedy	2*n*=2*x*=18	Helena Storchova

Supplementary Table S2. SSRs in the mitochondrial genomes of *Chenopodium* species.

Species	Accession	Mono	Di	Tri	Tetra	Penta	Hexa	Total
C. quinoa	0654	7	4	8	25	4	0	48
C. quinoa	Cherry Vanilla	7	4	8	25	4	0	48
C. quinoa	Chucapaca	9	4	8	25	4	0	50
C. quinoa	CICA-17	7	4	8	25	4	0	48
C. quinoa	G-205-95DK	8	4	8	25	4	0	49
C. quinoa	Ku-2	8	4	8	25	4	0	49
C. quinoa	Kurmi	7	4	8	25	4	0	48
C. quinoa	Ollague	7	4	8	25	4	0	48
C. quinoa	Pasankalla	8	4	8	25	4	0	49
C. quinoa	PI 614886	9	4	8	25	4	0	50
C. quinoa	PI 634921	9	4	8	25	4	0	50
C. quinoa	Real	7	4	8	25	4	0	48
C. quinoa	Regalona	8	4	8	25	4	0	49
C. quinoa	Salcedo INIA	7	4	8	25	4	0	48
C. berlandieri subsp. *nuttalliae*	PI 568156	7	4	8	25	3	0	47

C. berlandieri var. *boscianum*	BYU 937	7	4	7	24	4	0	46
C. berlandieri var. *macrocalycium*	PI 666279	7	4	8	25	4	0	48
C. berlandieri var. *sinuatum*	Ames 33013	9	4	7	25	4	0	49
C. berlandieri var. *zschackei*	BYU 1314	6	4	8	25	4	0	47
C. hircinum	BYU 1101	8	3	8	25	4	0	48
C. hircinum	BYU 566	8	4	8	25	4	0	49
C. pallidicaule	Ames 13221	8	4	5	21	4	0	42
C. suecicum	328/6	11	5	6	17	6	0	45

Supplementary Table S3. SSRs in the chloroplast genomes of *Chenopodium* species.

Species	Accession	Mono	Di	Tri	Tetra	Penta	Hexa	Total
C. quinoa	0654	5	3	1	6	1	0	16
C. quinoa	Cherry Vanilla	5	2	1	6	1	0	15
C. quinoa	Chucapaca	4	3	1	6	1	0	15
C. quinoa	CICA-17	5	3	1	6	1	0	16
C. quinoa	G-205-95DK	5	3	1	6	1	0	16
C. quinoa	Ku-2	5	3	1	6	1	0	16
C. quinoa	Kurmi	5	3	1	6	1	0	16
C. quinoa	Ollague	5	3	1	6	1	0	16
C. quinoa	Pasankalla	5	3	1	6	1	0	16
C. quinoa	PI 614886	4	2	1	6	1	0	14
C. quinoa	PI 634921	4	1	1	6	1	0	13
C. quinoa	Real	5	3	1	6	1	0	16
C. quinoa	Regalona	5	3	1	6	1	0	16
C. quinoa	Salcedo INIA	5	3	1	6	1	0	16
C. berlandieri subsp. *nuttalliae*	PI 568156	5	3	1	6	1	0	16
C. berlandieri var. *boscianum*	BYU 937	5	1	1	6	1	1	15
C. berlandieri var. *macrocalycium*	PI 666279	3	3	1	6	1	0	14
C. berlandieri var. *sinuatum*	Ames 33013	4	3	1	6	1	0	15
C. berlandieri var. *zschackei*	BYU 1314	4	3	1	6	0	2	16
C. hircinum	BYU 1101	4	3	1	6	1	0	15
C. hircinum	BYU 566	4	3	1	6	1	0	15
C. pallidicaule	Ames 13221	6	1	1	6	0	0	14
C. suecicum	328/6	7	1	1	7	0	0	16
